# Efficacy study of *Prunus amygdalus* (almond) nuts in scopolamine-induced amnesia in rats

**DOI:** 10.4103/0253-7613.66841

**Published:** 2010-06

**Authors:** Kirti S. Kulkarni, S.B. Kasture, S.A. Mengi

**Affiliations:** Department of Pharmacology, C. U. College of Pharmacy, SNDT Women’s University, Santacruz (W), Mumbai, India; 1 Department of Pharmacology, Sanjeevani College of Pharmaceutical Education and Research, Kopargaon, Dist. Ahmednagar, Maharashtra, India

**Keywords:** Alzheimer’s disease, amnesia, *Prunus amygdalus*, scopolamine

## Abstract

**Objective:**

Cognitive disorders such as amnesia, attention deficit and Alzheimer’s disease are emerging nightmares in the field of medicine because no exact cure exists for them, as existing nootropic agents (piractam, tacrine, metrifonate) have several limitations. The present study was undertaken to investigate the effect of *Prunus amygdalus* (PA) nuts on cognitive functions, total cholesterol levels and cholinesterase (ChE) activity in scopolamine-induced amnesia in rats.

**Materials and Methods:**

The paste of PA nuts was administered orally at three doses (150, 300 and 600 mg/kg) for 7 and 14 consecutive days to the respective groups of rats. Piracetam (200 mg/kg) was used as a standard nootropic agent. Learning and memory parameters were evaluated using elevated plus maze (EPM), passive avoidance and motor activity paradigms. Brain ChE activity and serum biochemical parameters like total cholesterol, total triglycerides and glucose were evaluated.

**Results:**

It was observed that PA at the above-mentioned doses after 7 and 14 days of administration in the respective groups significantly reversed scopolamine (1 mg/kg i.p.)-induced amnesia, as evidenced by a decrease in the transfer latency in the EPM task and step-down latency in the passive avoidance task. PA reduced the brain ChE activity in rats. PA also exhibited a remarkable cholesterol and triglyceride lowering property and slight increase in glucose levels in the present study.

**Conclusion:**

Because diminished cholinergic transmission and increase in cholesterol levels appear to be responsible for the development of amyloid plaques and dementia in Alzheimer patients, PA may prove to be a useful memory-restorative agent. It would be worthwhile to explore the potential of this plant in the management of Alzheimer’s disease.

## Introduction

Alzheimer’s disease (AD) is a progressive neurodegenerative disorder that is slow in onset but, ultimately, leads to dementia, unusual behavior, personality change and, ultimately, death.[1] Formation of memory is the most complex process and involves multiple neuronal pathways and neurotransmitters. It is well known that the cholinergic neuronal system plays an important role in learning and memory in humans and animals.[2] This is the therapeutic rational behind the use of nootropic agents such as piracetam, various piracetam analogues like oxiracetam, aniracetam[3] and metrifonate.[4] However, the adverse effects associated with these agents have limited their use.[5] The Indian system of medicine is replete with medicinal plants claimed to promote learning, memory and intelligence.[6] The plants like *Acorus calamus, Bacopa monniera, Tinospora cordifolia* and *Withania somnifera*[7] have been investigated for their effect on cognitive function of the brain. These plants have been grouped under the general class of *Medhya rasayanas* in the classical texts of Ayurveda, i.e., substances/agents that counter the degenerative changes associated with ageing and are beneficial in promoting intellect.[8]

*Prunus amygdalus* Batsch (Rosaceae; PA) is a small tree indigenous to regions around the Mediterranean Sea. The edible portion of PA is its nuts, commonly known as almonds or badam, and is a popular nutritious food.[9] Nuts of PA are found to possess various pharmacological properties, such as antistress,[10] antioxidant,[11] immunostimulant,[12] lipid lowering[13] and laxative.[8] The nuts of PA are mentioned as *medhyarasayana* (nootropic agent) in the classical Ayurvedic texts[8] and are also used in folklore practice. However, there is a lacuna with respect to the scientific evaluation of nuts of PA in preclinical animal models of learning and memory. In the light of the above, the present study was undertaken to investigate the effect of PA on cognitive function and brain cholinesterase (ChE) activity in scopolamine-induced amnesia in rats.

Neurochemical analysis of Alzheimer’s disease has revealed that there is a marked reduction in the acetylcholine (ACh) content of the cortical and hippocampal regions of the human brain. Centrally acting antimuscarinic drugs (like scopolamine) impair learning and memory of rats[14] and human beings.[15] The amnesia induced by scopolamine represented the main symptom of Alzheimer’s disease, and piracetam reversed this effect in rodents.[16] Further, recent studies have indicated that there is a connection between high cholesterol levels and the incidence of Alzheimer’s disease. It has been proposed that increased cholesterol levels could predispose a greater level of amyloid plaques.[17] Hence, the serum total cholesterol and triglyceride levels were also assessed in the present study. The Indian system of medicine is based on complete well being and, hence, the duration of treatment is prolonged to provide assured benefits. Therefore, the present study is conducted in two sets to evaluate the effect of prolonged treatment against scopolamine-induced amnesia. In the first set, treatment was provided for 7 days to rats whereas in the second set, treatment was provided for 14 days to rats, respectively.

## Materials and Methods

### Animals

Male albino rats of Wistar strain were used in this study. Initially, their weights were 150–180 g. The rats were allowed free access to food (Amrut Laboratory Animal Feeds, Bangalore, India) and water. Animals were housed in groups of three to four per cage and were kept under controlled room temperature (24±2°C) in a 12-h light-dark cycle. The experiment was conducted in a noise-free environment between 8.00 and 12.00 am. Prior approval was obtained from the Institutional Animal Ethics Committee, C. U. Shah College of Pharmacy, Mumbai, India, for conducting this study.

### Acute toxicity study

The acute toxicity was performed according to the OECD 423 guidelines.[18] The paste, at the dose of 5, 50, 300 and 2000 mg/kg body weight, was administered to the rats and they were subsequently observed closely for the first 4 h for any untoward symptoms such as tremors, convulsions, exophthalmus, salivation, diarrhea and lethargy followed by observation for a further 14 days. At the end of the experimental period, the animals were observed for any changes in behavioral pattern and mortality.

### Chemicals

The chemicals used in this study were obtained from the following drug houses. Scopolamine, Acetylcholine, metrifonate (Sigma Laboratories, Mumbai, Maharashtra, India.), piracetam (UCB India Ltd., Mumbai, Maharashtra, India.), simvastatin (Krebs Biochemicals and Industries Limited, Hyderabad, India), sodium dihydrogen phosphate, disodium hydrogen phosphate (Hi Media, Mumbai, India) and cholesterol, triglycerides, total proteins and glucose diagnostic kit (Transasia Biomedicals, Mumbai, India).

### Drugs administration

The nuts of PA were purchased from the local market and authenticated at the Botany Department of Agharkar Research Institute, Pune, India. The fine paste of the PA nuts was prepared in distilled water and sonicated for 20 min to obtain a fine suspension. Then, the paste was administered orally to the rats at three doses of 150, 300 and 600 mg/kg/day. The above dose levels were selected by the conversion of conventional human dose into animal dose. The human dose of PA was five to six nuts daily (approximately 6 g).

PA was administered at the same time on each day (i.e., 8.00–9.00 am) for 7 and 14 days, respectively.

### Experimental design

The experimental design was planned such that the effect of PA at doses of 150, 300 and 600 mg/kg could be evaluated after 7 and 14 days against scopolamine-induced amnesia. For this purpose, the rats were divided into two sets of eight groups each (16 groups). The treatment period for animals of set I and set II was 7 and 14 days, respectively. At the end of the treatment period, all the animals were subjected to scopolamine (1 mg/kg i.p.)[14] 60 min after the drug administration, except the first group of each set, which served as a vehicle control. The cognitive paradigms were evaluated 45 min after the scopolamine administration using the elevated plus maze (EPM) and passive avoidance models. This was termed as the acquisition trial (AT), which corresponds to learning. Further, the retention trial (RT) was carried out after 24 h of scopolamine administration. In the RT, the above-mentioned parameters were re-assessed as an index of memory. Additionally, locomotor activity was assessed using an actophotometer. Further, serum biochemical parameters such as serum total cholesterol, triglycerides and glucose were evaluated and then the animals were euthanized by cervical decapitation, and the brains were isolated to evaluate the anticholinesterase (ChE) activity of PA.

### Exteroceptive behavioral models

#### EPM

The EPM[19,20] served as the exteroceptive behavioral model (wherein the stimulus existed outside the body) to evaluate learning and memory in rats. The plus maze apparatus consisted of two open (50 cm × 10 cm × 40 cm) and two enclosed arms (50 cm × 10 cm × 40 cm), with an open roof, arranged such that the two open arms were opposite each other. The maze was elevated to a height of 50 cm from the ground to measure the anxiety index in rats.

On the 7^th^ and 14^th^ days, respectively, each rat was placed at the end of an open arm, facing away from the central platform. Transfer latency (TL) was considered as the time taken by the rats to move into any one of the closed arms with all four legs. TL was recorded. If the rat did not enter into one of the closed arms within 180 s, it was gently pushed into one of the two closed arms and the TL was assigned as 180 s. The rat was allowed to explore the maze for 15 s and then returned to its home cage. Twenty-four hours later, i.e. on days 8 and 15, TL was recorded again. The measurements of TL on days 7 and 14 served as parameters for acquisition and those on days 8 and 15 served as parameters for retention of memory.

### Passive shock avoidance paradigm

Passive avoidance,[19,20] based on negative reinforcement, was recorded to examine the long-term memory. The apparatus consisted of a box (27 cm × 27 cm × 27 cm) having three walls of wood and one wall of Plexiglas, featuring a grid floor (made up of 3 mm stainless-steel rods set 8 mm apart), with a wooden platform (10 cm × 7 cm × 1.7 cm) in the center of the grid floor. The box was illuminated with a 15 W bulb during the experimental period. Electric shock was delivered to the grid floor. The rats were initially trained as follows: each rat was placed on a wooden platform set in the centre of the grid floor. When the rat stepped down and placed its paw on the grid floor, shock (foot shock: 50 Hz; 1.5 mA; 1 s) was delivered and the step-down latency (SDL) was recorded. SDL is defined as the time taken by the rat to step down and place all four paws on the grid floor. Rats showing SDL in the range of 2-15 s during the training session were taken for the acquisition and the retention tasks. The acquisition task was carried out 90 min after the training session. During the acquisition test, animals were removed from the shock-free zone if they did not step down for a period of 60 s. Retention was tested after 24 h in a similar manner, except with an upper cut-off time of 180 s.

### Locomotor activity[21]

The locomotor activity (horizontal activity) was measured using an actophotometer. Each rat was placed individually in the actophotometer for 5 min and the basal activity score was obtained. Subsequently, the animals were divided into 16 groups, each group consisting of six animals. The respective treatments were administered and, after 60 min, the rats were again placed in the actophotometer for recording the activity score as described earlier.

### Biochemical Estimations

The blood was withdrawn from the retroorbital plexus of the rats under mild ether anesthesia on day 0 (before starting the drug administration), day 7 (for animals of set I) and day 14 (for animals of set II). The animals were fasted for 18 h prior to blood withdrawal. The blood was collected and centrifuged to separate serum for estimation of biochemical parameters. The serum total cholesterol, triglyceride and glucose levels were analyzed by an enzymatic colorimetric method using commercially available kits (ERBA Diagnostics, Mannheim, Germany, Transasia Bio-Medicals Ltd., Mumbai, India) and an ERBA Chem. 5 semiautoanalyzer (ERBA Diagnostics, Mannheim, Germany).[17,20]

### Estimation of ACh Levels in the Brain by Quantifying ChE Inhibition

After assessing the learning and memory paradigms in scopolamine-induced amnesia, rats from each group were euthanized by cervical decapitation. The whole brain was immediately removed and chilled in ice-cold phosphate buffer. After washing in ice-cold phosphate buffer, the brains were homogenized in 5 ml of phosphate buffer in a glass TEFLON homogenizer. The brain homogenate was then evaluated for enzyme activity using Augustinsson’s method of analysis.[22]

### Statistical Analysis

Statistical analysis was performed by one-way analysis of variance (ANOVA) followed by Dunnetts *t*-test. Values are expressed as mean ± SEM and *P* <0.05 was considered to be significant.

## Results

### Acute toxicity profile

The rats treated with the paste of PA, 5–2,000 mg/kg, p.o., exhibited normal behavior. They were alert, with normal grooming, touch response and pain response. There was no sign of passivity, stereotypy and vocalization. Their motor activity and secretory signs were also normal. The animals showed no signs of depression. Alertness, limb tone and grip strength as well as the gait of the animals were normal. The paste of PA was found to be safe up to a dose 2,000 mg/kg in rats.

### Exteroceptive behavioral models

#### EPM

The effect of the vehicle, scopolamine control, PA (150, 300 and 600 mg/kg) and piracetam (120 mg/kg) were evaluated at the end of days 7 and 14. The scopolamine (1 mg/kg) control group showed a significant (*P* < 0.01) increase in TL values on the acquisition as well as on the retention days as compared with vehicle control rats, indicating impairment in learning and memory. In the AT on day 7 for set I and on day 14 for set II, the PA at dose levels 150, 300 and 600 mg/kg demonstrated decrease in the TL as compared to the scopolamine control group. The results obtained were found to be statistically significant (*P* < 0.01). In the RT on day 8 for set I and day 15 for set II, the PA at the dose levels 150, 300 and 600 mg/kg demonstrated a significant (*P* < 0.01) decrease in the TL as compared to the scopolamine control group. Piracetam (120 mg/kg p.o.) exhibited marked decrease (*P* < 0.01) in TL in comparison with the scopolamine control group. However, PA at the dose levels 300 and 600 mg/kg showed a decrease in the TL, which is comparable to that shown by piracetam (*P* < 0.01) [Table 1].

**Table 1:** Effect of the paste of *Prunus amygdalus* on transfer latency (elevated plus maze paradigm) in scopolamine-induced amnesia in rats

*Treatment groups*	*TL on acquisition day (sec)*		*TL on retention day (sec)*	
	*7 day*	*14 day*	*8 day*	*15 day*
Vehicle control	95.17 ± 14.92	39.83 ± 5.89	55.33 ± 8.77	23.83 ± 04.13
Scopolamine hydrobromide (1)	143.67 ± 17.2*	118.8 ± 11.03††	128.33 ± 19.53**	118.83 ± 26.13††
*Prunus amygdalus* (150) + scopolamine (1)	65.17 ± 7.98^a^	32.17 ± 7.71^b^	40.16 ± 8.16^a^	12.67 ± 2.5^b^
*Prunus amygdalus* (300) + scopolamine (1)	55.5 ± 4.75^a^	17.33 ± 4.73^b^	23.83 ± 3.69^a^	10.17 ± 2.33^b^
*Prunus amygdalus* (600) + scopolamine (1)	30.5 ± 6.67^a^	15.33 ± 3.05^b^	16.83 ± 1.74^a^	3.5 ± 0.34^b^
Piracetam (120) + scopolamine (1)	52.17 ± 9.85^a^	26.67 ± 3.60^b^	34.17 ± 5.21^a^	7.33 ± 2.13^b^
Simvastatine (0.5) + scopolamine (1)	113.17 ± 12.92^a^	86.17 ± 13.5††^b^	82.67 ± 16.72^a^	77.17 ± 5.78^b^

Values are expressed as mean ± SEM at *n* = 6; One-way ANOVA followed by Dunnett’s test;

* *P* <0.05;

†† *P* <0.01 versus the control group (14 days);

** *P* <0.01 versus control group (7 days);

a *P* <0.01 compared to the scopolamine (1 mg/kg)-administered group on day 7;

b *P* <0.01 compared to the scopolamine (1 mg/kg)-administered group on day 14

### Passive shock avoidance paradigm

Scopolamine hydrobromide (1 mg/kg i.p.) decreased SDL on the AT and RT training, indicating impairment of memory. There is a slight increase in SDL after the administration of PA (150, 300 and 600 mg/kg p.o.) for 7 days as compared with the scopolamine control group on the AT and RT, indicating improvement in learning and memory of rats. However, in the AT, PA at the dose levels 150 and 300 mg/kg p.o. increased SLD, which is comparable to standard piracetam, but failed to exhibit a significant change when compared with the scopolamine control after 14 days of administration. However, PA (150, 300 and 600 mg/kg) was found to significantly (*P* < 0.05) decrease the SDL on RT when compared with the scopolamine control after 14 days of administration [Table 2]. PA (150, 300 and 600 mg/kg) after 14 days of administration showed a slight decrease in the SDL on acquisition day as compared to the groups that received the day 7 administration. However, on the retention day, PA (150, 300 and 600 mg/kg) after 14 days of administration showed a marked increase in the SDL as compared to the day 7 administration groups.

**Table 2:** Effect of the paste of *Prunus amygdalus* on step-down latency (passive avoidance paradigm) in scopolamine-induced amnesia in rats

*Treatment groups*	*SDL on acquisition day (sec)*		*SDL on retention day (sec)*	
	*7 day*	*14 day*	*8 day*	*15 day*
Vehicle control	2.67 ± 0.67	1.83 ± 0.47	3.16 ± 0.79	2.16 ± 0.60
Scopolamine hydrobromide (1)	2.83 ± 0.47	1.17 ± 0.17	1.67 ± 0.49	1.83 ± 0.30
*Prunus amygdalus* (150) + scopolamine (1)	3.67 ± 1.41	3.00 ± 0.58	4.00 ± 0.85	6.00 ± 1.51^b^
*Prunus amygdalus* (300) + scopolamine (1)	2.00 ± 0.51	3.16 ± 0.87	4.17 ± 1.35	6.67 ± 2.35^b^
*Prunus amygdalus* (600) + scopolamine (1)	2.00 ± 0.68	3.33 ± 0.72	5.00 ± 1.49	5.67 ± 1.89^b^
Piracetam (120) + scopolamine (1)	2.5 ± 0.76	3.5 ± 0.72	5.83 ± 1.25	7.00 ± 2.13^b^
Simvastatine (0.5) + scopolamine (1)	1.67 ± 0.43	1.83 ± 0.60	2.58 ± 0.40	2.83 ± 0.48

Values are expressed as mean ± SEM at *n* = 6; One-way ANOVA followed by Dunnett’s test; ^*^P <0.05 versus control group (7 days); ^a^*P* <0.01 compared to the scopolamine (1 mg/kg)-administered group on the 7^th^ day; ^††^*P* <0.01 versus control group (14 days);

b *P* <0.01 compared to the scopolamine (1 mg/kg)-administered group on day 14^th^ day

### Locomotor activity

The paste of PA at doses of 150, 300 and 600 mg/kg p.o. did not produce any significant reduction in locomotor activity as compared to the control animals receiving only the vehicle. Also, the scopolamine control group and the piracetam-treated group failed to produce any significant effect on locomotor activity as compared to the control. This result indicates that the scopolamine injection did not affect the general locomotor activities of the rat, but gave rise to learning disabilities in them [Table 3].

**Table 3:** Effect of the paste of *Prunus amygdalus* on serum total cholesterol, triglycerides and glucose levels in scopolamine-induced amnesia in rats

*Treatment groups*	*Serum total cholesterol (mg/dl)*		*Serum triglycerides (mg/dl)*		*Serum glucose (mg/dl)*	
	*8 day*	*15 day*	*8 day*	*15 day*	*8 day*	*15 day*
Vehicle control	67.04 ± 5.33	56.52 ± 5.61	82.21 ± 8.18	82.88 ± 12.51	90.32 ± 7.16	90.72 ± 4.14
Scopolamine hydrobromide (1)	76.88 ± 6.52	82.78 ± 5.78	112.71 ± 21.90	93.97 ± 13.24^b^	71.76 ± 6.22	78.51 ± 3.23
*Prunus amygdalus* (150) + scopolamine (1)	62.78 ± 4.78	51.07 ± 1.93^b^	77.79 ± 24.34^a^	70.84 ± 13.27^b^	86.07 ± 2.71	90.62 ± 4.34
*Prunus amygdalus* (300) + scopolamine (1)	58.61 ± 5.76	52.21 ± 3.78^b^	67.71 ± 4.34^a^	63.21 ± 13.42^b^	91.92 ± 9.13	99.96 ± 9.20
*Prunus amygdalus* (600) + scopolamine (1)	57.05 ± 4.97	48.60 ± 2.14^b^	70.63 ± 6.58^a^	57.86 ± 10.69^b^	92.93 ± 7.23	101.54 ± 8.58
Piracetam (120) + scopolamine (1)	58.80 ± 4.78	45.13 ± 3.29^b^	54.81 ± 4.20^a^	50.37 ± 3.0^b^	81.98 ± 9.53	85.69 ± 12.92
Simvastatin (0.5) + scopolamine (1)	58.62 ± 4.79	42.35 ± 2.25^b^	52.38 ± 3.61^a^	39.54 ± 3.24^b^	71.85 ± 5.32	82.95 ± 8.56

Values are expressed as mean ± SEM at *n* = 6; One-way ANOVA followed by Dunnett’s test; ^*^P <0.05 versus control group (7 days); ^††^*P* <0.01 versus control group (14 days);

b *P* <0.01 compared to the scopolamine (1 mg/kg)-administered group on day 14^th^ day; Simvastatin (0.5 mg/kg, p. o.) was used as the standard drug

a *P* <0.01 compared to the scopolamine (1 mg/kg)-administered group on day 7^th^ day;

### Biochemical estimations

Pretreatment with PA at doses of 150, 300 and 600 mg/kg p.o. for 7 days showed marked reduction in serum total cholesterol, but failed to exhibit a significant change when compared with the scopolamine control. However, pretreatment with PA at doses of 150, 300 and 600 mg/kg p.o. for 7 days exhibited a significant reduction (*P* < 0.01) in the serum triglyceride levels when compared with the scopolamine control group. The animals receiving PA at doses of 150, 300 and 600 mg/kg for 14 days showed a significant (*P* < 0.01) reduction in the total cholesterol and triglycerides, which was similar to that of simvastatin. The animals receiving PA at doses of 150, 300 and 600 mg/kg for 7 days (for set I) and 14 days (for set II) showed a slight increase in the glucose levels [Table 4].

**Table 4:** Effect of the paste of *Prunus amygdalus* on the locomotor activity in scopolamine-induced amnesia in rats

*Treatment groups*	*Activity score*		*Activity score*	
	*0 day*	*7 day*	*0 day*	*14 day*
Vehicle control	156.00 ± 16.40	161.00 ± 13.41	158.33 ± 13.79	174.33 ± 6.44
Scopolamine hydrobromide (1)	141.33 ± 11.00	149.67 ± 10.64	137.5 ± 12.30	158.00 ± 12.12
*Prunus amygdalus* (150) + scopolamine (1)	155.33 ± 17.72	133.33 ± 13.58	145.17 ± 11.41	145.00 ± 13.09
*Prunus amygdalus* (300) + scopolamine (1)	163.83 ± 18.12	157.33 ± 17.76	160.50 ± 18.24	152.00 ± 9.94
*Prunus amygdalus* (600) + scopolamine (1)	199.17 ± 28.03	167.50 ± 12.76	164.00 ± 22.20	160.50 ± 9.82
Piracetam (120) + scopolamine (1)	165.33 ± 16.63	172.50 ± 22.57	177.50 ± 22.59	192.50 ± 22.38
Simvastatine (0.5) + scopolamine (1)	175.33 ± 11.63	175.83 ± 21.34	189.17 ± 18.14	168.33 ± 15.00

Values are expressed as mean ± SEM at *n* = 6; The differences in mean were not significant at *P* <0.05; One-way ANOVA followed by Dunnett’s test

### Estimation of AChE activity in the brain

**Figure 1:**
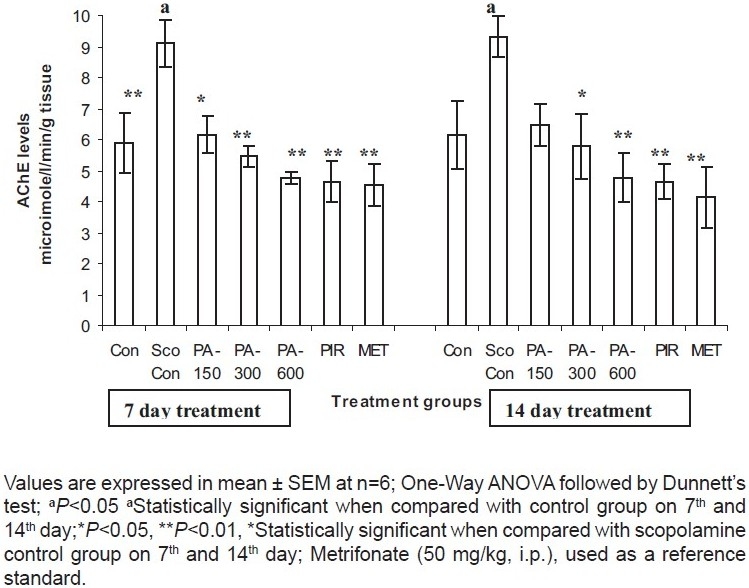
Estimation of the acetylcholinesterase activity in rat brain homogenate in scopolamine induced amnesia.

The AChE activity of the whole brain was markedly reduced (*P* < 0.05) after metrifonate (50 mg/kg i.p.) treatment. Piracetam (120 mg/kg p.o.) and PA (150, 300 and 600 mg/kg p.o.) significantly (*P* < 0.01) reduced the levels of AChE, which is considered as an indicator of inhibition of AChE activity in the rat brain after 7 days of treatment. However, on 14 days of treatment, piracetam (120 mg/kg p.o.) and PA (300 and 600 mg/kg p.o.) significantly (*P* < 0.01) reduced the levels of AChE, which is considered as an indicator of inhibition of AChE activity [Figure 1].

## Discussion

Alzheimer’s disease is a neurogenerative disorder associated with a decline in cognitive abilities.[23] Despite the severity and high prevalence of this disease, the allopathic system of medicine is yet to provide a satisfactory antidote. Hence, the present study focuses on exploration of the memoryenhancing activity of the paste of PA nuts in a scopolamineinduced amnesia rat model.

The present study suggests that PA possesses memoryenhancing activity in view of its facilitatory effect on the retention of spatial memory in scopolamine-induced amnesia. There is a decrease in the TL, i.e. rats were able to locate the dark zone immediately after exposure to the open arm in the EPM paradigm, which is an indicator of cognition improvement.

In case of the passive avoidance paradigm, the SDL is increased on administration of PA. This suggests that the animal has the retention of memory of the shock once entered in the shock-free zone. The long-term administration of the PA paste (14-day administration) exhibited pronounced effect in the reversal of the scopolamine-induced amnesia in case of the passive avoidance paradigm as compared to the 7-days administration.

It is well known that cholinergic neuronal systems play an important role in the cognitive deficits associated with AD, ageing and neurodegenerative diseases.[24] In our study, metrifonate *per se* (50 mg/kg p.o.) significantly reduced brain AChE activity. Piracetam (120 mg/kg p.o.) and PA (150, 300 and 600 mg/kg p.o.) significantly lowered this activity, indicating the stimulating actions of these drugs on the cholinergic system. Hence, the memory-enhancing effect of the PA can be attributed to its anti-ChE activity.

The main histological features of Alzheimer’s disease include extracellular protein deposits, termed as amyloid beta (AB) plaques, AB deposits in blood vessels and intraneuronal neurofibrillary tangles.[25] Abnormal accumulation of cholesterol levels increase AB in cellular and most animal models of AD, and drugs that inhibit cholesterol synthesis lower AB in these models.[26] Interestingly, animals that were treated with PA for 14 days showed a marked (*P* < 0.01) reduction in cholesterol and triglyceride levels as compared to the scopolamine control groups in addition to their nutritional value (as glucose levels were slightly higher when compared to controls).

The probable active constituent of the PA was omega 3 fatty acids. Gas chromatography mass spectrometry (GCMS) evaluations confirmed the presence of omega 3 fatty acids in PA oil. GCMS analysis was carried out by the Department of SAIF IITB on request. Thus, the presence of omega 3 fatty acids in the nuts may be responsible for the above-mentioned activities.[26] The peer review of the above findings suggests that PA may prove to be a useful anti-Alzheimer agent.

Therefore, the memory-improving activity of PA may be attributed to the anti-AChE, procholinergic, cholesterol reduction, neuroprotective and nutritive properties of the PA nuts. Hence, PA may be used in delaying the onset and reducing the severity of Alzheimer’s disease. However, further investigations are warranted to explore the possible involvement of other neurotransmitters such as glutamate, Gamma aminobutyric acid (GABA) and catecholamines, responsible for the memory-improving property of PA.

## Conclusion

In the present study, we observed that PA lowered serum cholesterol in rats, elevated Acetylcholine (ACh) level in the brain and, ultimately, improved the memory (spatial and avoidance) of rats on administration for 7 days and 14 days, respectively. In the light of the above, it may be worthwhile to explore the potential of this plant in the management of cognitive dysfunction.
